# The incidence and influencing factors of type 2 diabetes mellitus in Chinese adults in 10 provinces: a prospective cohort study

**DOI:** 10.1186/s12889-025-22584-9

**Published:** 2025-07-04

**Authors:** Shujuan Li, Tingfang Ai, Run Zhang, Xiaoqing You, Jianhong Li

**Affiliations:** 1 Chinese Field Epidemiology Training Program, Chinese Center for Disease Control and Prevention, Beijing, 100050 China; 2 National Center for Chronic and Noncommunicable Disease Control and Prevention, Chinese Center for Disease Control and Prevention, Beijing, 100050 China; 3 National Institute for Nutrition and Health, Chinese Center for Disease Control and Prevention, Beijing, 100050 China

**Keywords:** Diabetes mellitus, Incidence, Influencing factors, Prospective cohort study, China

## Abstract

**Background:**

Diabetes mellitus (DM) is an important public health problem that seriously affects the health of Chinese residents. Exploring the incidence of DM and its influencing factors in Chinese adults could help develop prevention and control strategies and measures. However, there was few national cohort study on DM incidence and its influencing factors in China. Based on the national representative data, we investigated the incidence and influencing factors of DM in Chinese adults.

**Methods:**

Based on 2010 China Chronic Disease and Risk Factors Surveillance (CCDRFS), a follow-up survey was conducted in 20 monitoring points from ten provinces of China in 2016–2017. Incident cases of DM were identified through questionnaires. Influencing factors for DM were collected at baseline using questionnaires, physical measurements and laboratory tests. Cox regression models were used to analyze the influencing factors of DM. Sensitivity analysis was conducted to confirm the associations between the influencing factors and DM.

**Results:**

A total of 7048 people were included in this study. During the follow-up period, 699 DM patients were diagnosed (313 males and 386 females), with a total follow-up of 40022.82 person-years. The incidence density of DM was 17.5/1000 person-years. The Cox regression results showed that age 45–59 years (HR = 1.32, 95% CI: 1.10 ~ 1.59) and ≥ 60 years (HR = 1.48, 95% CI: 1.17 ~ 1.88), divorce/widowhood (HR = 1.45, 95% CI: 1.15 ~ 1.82), overweight (overweight with no central obesity: HR = 1.26, 95% CI: 1.03 ~ 1.54; overweight and central obesity: HR = 1.62, 95% CI: 1.34 ~ 1.97), hypertension (HR = 1.35, 95% CI: 1.15 ~ 1.59), hypertriglyceridemia (HR = 1.57, 95% CI: 1.28 ~ 1.91), high LDL-C (HR = 1.20, 95% CI: 1.02 ~ 1.40), sitting > 6 h for men (HR = 1.51, 95% CI: 1.11 ~ 2.06), and family history (HR = 1.55, 95% CI: 1.17 ~ 2.06) were risk factors for DM.

**Conclusions:**

The incidence of DM for Chinese adults from 10 provinces was 17.5/1000 person-years. Age > 44 years, divorced/widowed, overweight, central obesity, hypertension, dyslipidemia, a sedentary lifestyle and a family history of diabetes were risk factors for DM. Comprehensive preventive measures centered on a healthy diet, adequate physical exercise, and maintaining a healthy weight should be further promoted to prevent diabetes in China.

## Introduction

Diabetes mellitus (DM) has become an important threat to human health worldwide, which not only causes serious health burdens, including a variety of complications, such as renal failure, blindness, cardiovascular disease and death but also heavy economic burdens [1]. According to estimations of the International Diabetes Federation (IDF), 536,600 thousand people suffer from diabetes worldwide, and this number will reach 642,800 in 2030 and 783,700 in 2045 [2]. Diabetes is responsible for 6.7 million deaths in 2021–1 every 5 s, and diabetes has caused at least USD 966 billion dollars in health expenditures—a 316% increase over the last 15 years [2].

In China, the prevalence of diabetes has increased significantly over the past 30 years. China has become the country with the largest number of patients with diabetes [2]. In 1980, the prevalence of diabetes in China was 0.67% [3]. The prevalence of diabetes among people aged 18 years and over was 10.4% in 2013 [4], and the prevalence of diabetes and prediabetes in China were 12.4% and 38.1%, respectively, in 2018 according to CCDRFS [5].

Over 90% of people with diabetes have type 2 diabetes, which is caused by socioeconomic, demographic, environmental, and genetic factors [2]. With the acceleration of urbanization in China, significant changes in socioeconomic-related environments, dietary structure and patterns, and lifestyles tend to lead to diabetes [6]. The dietary structure of Chinese residents, which includes more fat, and lifestyles with less physical activity, lead to an increase in overweight and obesity among residents [7]; at the same time, the aging of the population has led to an increase in the elderly population, which has both led to an increase in the prevalence of diabetes among Chinese residents.

Keeping track of diabetes incidence and the influencing factors of diabetes occurrence is important for developing targeted control and prevention strategies. Currently, there was few national cohort study on diabetes incidence and its influencing factors in China. In this study, based on data from 10 provinces of 2010 CCDRFS, we conducted a follow-up survey from 2016 to 2017 to explore the incidence of adult diabetes in China and its influencing factors.

## Methods

### Research subjects

This study is a prospective cohort study to investigate the DM incidence and its association with influencing factors. The baseline data was from 2010 CCDRFS. The follow-up survey was taken in 20 surveillance points from 10 provinces of 2010 CCDRFS. The 10 provinces including Hebei, Jilin, Heilongjiang, Zhejiang, Jiangxi, Henan, Hunan, Sichuan, Guizhou, and Shaanxi. For each province, 2 monitoring points (1 in urban areas and 1 in rural areas) were chosen as the follow-up survey points. People who met the requirements (having normal blood sugar) at the baseline were selected as survey subjects. The follow-up survey was conducted from 2016 to 2017, and 7048 people were ultimately followed up. This study was approved by National Center for Chronic and Noncmmunicable Disease Control and Prevention, Chinese Center for Disease Control and Prevention (approval number 201524B). All the participants signed the informed consent form, and for the illiterate participants, the informed consent of their parents and/or legal guardians was obtained.

CCDRFS is designed to measure the epidemiology of chronic disease and associated risk factors by selecting a nationally representative sample of the general population every 3 years [5]. The 2010 CCDRFS was carried out at 162 disease surveillance points covering major geographic areas of 31 provinces, autonomous regions, and municipalities in mainland China [8]. Representative samples were selected using multistage stratified cluster sampling, with residents aged 18 and above living in the sample area as the survey subjects, and the sampling process were presented in Table 1 [8]. More detailed survey design information have been reported previously [8, 9].

**Table 1:** Sampling process of the survey participants in 2010 CCDRFS

Sampling Stage	Sample Allocation	Sampling Method
Stage 1	Randomly select 4 townships (streets, groups)	Proportional to size (PPS) cluster sampling
Stage 2	Randomly select 3 administrative villages (residential committees, units)	Proportional to size (PPS) cluster sampling
Stage 3	Randomly select 1 villager/resident group (at least 50 households)	Cluster random sampling
Stage 4	Randomly select 1 person from each household	KISH grid method

### Investigation content

Both the baseline and follow-up surveys included three parts: an inquiry survey, physical measurements, and laboratory tests [9, 10]. The inquiry survey was conducted in a face-to-face manner. The questionnaire content included basic personal information, lifestyle information (smoking, drinking, diet, physical activity level, etc.), chronic disease occurrence, diagnosis, and control status [10, 11]. The physical measurements included height, weight, waist circumference, and blood pressure. During laboratory testing, 3–6 ml of venous blood was collected from each survey subject after 10–12 h on an empty stomach, and another 1 ml of venous blood was collected after 2 h of taking 75 g of glucose. The detection indicators included fasting blood glucose (FPG), 2-hour post glucose load blood glucose, glycated hemoglobin, and blood lipids.

### Diagnostic criteria

Diabetes is defined as participants with a fasting plasma glucose level of 7.0 mmol/L or greater, a 2-hour plasma glucose level of 11.1 mmol/L or patients who had been diagnosed with diabetes by township or community hospitals and above according to the standard established by the World Health Organization (WHO) in 1999.

### Model construction

Before constructing the COX regression models, we conducted a literature review to identify variables that have been shown to be significant in DM risk factor studies [6, 12–14]. Based on the literature review and the existing surveillance data, a theoretical framework for DM influencing factors was developed. The framework includes five aspects: the demographic characteristics, regional status, personal health status, personal lifestyle, and family history of DM. Firstly, the incidence of DM according to different characteristics was analyzed. Then, the Cox regression models for all research subjects and for different genders were constructed to find the risk factors of DM. Sensitivity analysis was undertaken to confirm the associations between the influencing factors and DM, which was taken as followings: Cox regression was repeated after excluding 65 participants who had developed DM in the first 2 years of follow-up.

### Definition and grouping of risk factors

The influencing factors in this study were obtained from the baseline survey, including demographic characteristics (age, sex, marital status, and education level), regional status (urban or rural residents, residing in the eastern area, central area or western area of China), personal health status (hypertension, dyslipidemia, weight status), personal lifestyle (smoking, drinking, and physical activity), and family history. Urban and rural residents were defined by the participants’ habitual residence, which was classified by the criteria of the National Bureau of Statistics (NBS) [15]. The residences in the East, Middle and West Regions were classified according to the criteria of the NBS [16]. The weight status data included Body Mass Index (BMI) and central obesity. BMI was divided into low body weight (< 18.5 kg/m^2^), normal body weight (18.5 kg/m^2^~), overweight (24.0 kg/m^2^~), and obese (≥ 28.0 kg/m^2^) according to the Guidelines for the Prevention and Control of Overweight and Obesity in Chinese Adults [17]. Central obesity was defined as a waist circumference ≥ 90 cm in males or ≥ 80 cm in females [18]. Those with Systolic Blood Pressure (SBP) ≥ 140 mmHg (1 mmHg = 0.133 kPa) and/or Diastolic Blood Pressure(DBP) ≥ 90 mmHg or those who had been taking antihypertensive drugs in the past 2 weeks were defined as having hypertension [19]. Dyslipidemia was defined as Total Cholesterol (TC), high-density lipoprotein cholesterol (HDL-C), low-density lipoprotein cholesterol (LDL-C), and triglyceride (TG) levels exceeding the diagnostic criteria. TG ≥ 2.26 mmol/L indicates hypertriglyceridemia, TC ≥ 6.22 mmol/L indicates hypercholesterolemia, LDL-C ≥ 4.14 mmol/L indicates high LDL-C, and HDL-C < 1.04 mmol/L indicates low HDL-C [20]. For dietary status, red meat was divided in to ≤ 100 g/day and > 100 g/day; intake of vegetables and fruits was divided in to < 400 g/day and ≥ 400 g/day; fried food intake was divided into < 100 g/week and ≥ 100 g/week; and sugar-containing beverage intake was divided into < 300 ml/week and ≥ 300 ml/week. Regarding habits, adults who were still smoking during the survey were smokers. Current drinking was defined as drinking any purchased or homemade beverage containing ethanol (including Chinese liquor, beer, red wine, wine, fruit wine, yellow rice wine, highland barley wine, etc.) in the past 12 months [21]. Sleeping time was divided into < 7 h, 7–9 h, and > 9 h. The sitting time was divided into ≤ 3 h, 3.1–4 h, 4.1–6 h, and > 6 h. Family history was defined as the family history of first-degree relatives, that is, at least one of the parents, children or brothers and sisters of the study subject with diabetes.

### Statistical methods

SAS 9.4 was used for the data analysis. The characteristics of the follow-up and missing up population were described using the number of examples and composition ratio, and χ2 tests were used for intergroup comparisons. Cox regression models multivariate analyses between the DM and the influencing factors were used to calculate the hazard ratios (HRs) and 95% confidence intervals (CIs). Cox proportional hazards regression models were employed to conduct multivariate analyses between DM and its influencing factors, thereby calculating the hazard ratios (HRs) and their corresponding 95% confidence intervals (CIs). Differences at *P* < 0.05 were considered statistically significant.

## Results

### The baseline characteristics

The baseline characteristics of the study subjects were described in Table 2. Among the 6967 study participants, 2986 were males and 3981 were females, with an average age of 46.7 years, and 85.2% were married. The education levels were mostly primary school or below (43.2%) and junior high school (34.2%). The proportion in the central region was relatively high (48.9%), and 54.8% of the research subjects were from rural areas. The detection rates of overweight and obesity, central obesity, hypertension were 47.8%, 25.6%, 38.9% respectively. The detection rates of hypertriglyceridemia, hypercholesterolemia, high LDL-C and low HDL-C were 11.7%, 3.0%, 2.1% and 41.9%. For dietary behavior, the proportions of people with red meat intake > 100 g/day, vegetables and fruits intake < 400 g/day, fried food intake ≥ 100 g/week, and sugar-containing beverage intake ≥ 300 ml/week were 33.0%, 48.6%, 19.3% and 16.8%, respectively. The prevalence of current smoking and drinking habits was 33.9% and 38.6%, respectively, and the prevalence of sleeping for < 7 h and sitting for > 6 h was 18.4% and 17.9%, respectively. The percentage of patients with a family history of diabetes was 5.3%. In general, the study subjects exhibited distinct characteristics across different groups.

**Table 2:** Basic characteristics of the research subjects

Influencing factors	Male (*n* = 2986)	Female (*n* = 3981)	All (*n* = 6967)
Age, y			
18 ~ 44	1326 (44.4)	1784 (44.8)	3110(44.6)
45 ~ 59	1067 (35.7)	1542 (38.7)	2609(37.5)
≥ 60	593 (19.9)	655 (16.5)	1248(17.9)
Marital status			
Married	2511 (84.1)	3425 (86.0)	5936(85.2)
Unmarried	252 (8.4)	141 (3.5)	393(5.6)
Divorce/Widow	223 (7.5)	415 (10.4)	638(9.2)
Education level			
≤Primary school	1085 (36.3)	1925 (48.4)	3010(43.2)
Secondary school	1129 (37.8)	1256 (31.5)	2385(34.2)
High school	529 (17.7)	512 (12.9)	1041(14.9)
≥College or above	243 (8.2)	288 (7.2)	531(7.7)
Regions			
East	448 (15.0)	727 (18.3)	1175(16.9)
Middle	1479 (49.5)	1926 (48.4)	3405(48.9)
West	1059 (35.5)	1328 (33.4)	2387(34.3)
Urban/rural			
Urban	1317 (44.1)	1829 (45.9)	3146(45.2)
Rural	1669 (55.9)	2152 (54.1)	3821(54.8)
Overweight and obesity			
< 18.5	123 (4.1)	154 (3.9)	277(4.0)
18.5 ~ 23.9	1489 (49.9)	1865 (46.8)	3354(48.1)
24 ~ 27.9	977 (32.7)	1324 (33.3)	2301(33.0)
28.0~	397 (13.3)	638 (16.0)	1035(14.8)
Central obesity			
Yes	659 (22.1)	1124 (28.2)	1783(25.6)
No	2327 (77.9)	2857 (71.8)	5184(74.4)
Hypertension			
Yes	1198 (40.1)	1513 (38.0)	2711(38.9)
No	1788 (59.9)	2468 (62.0)	4256(61.1)
Hypertriglyceridemia			
Yes	426 (14.3)	387 (9.7)	813(11.7)
No	2560 (85.7)	3594 (90.3)	6154(88.3)
Hypercholesterolemia			
Yes	93 (3.1)	118 (3.0)	211(3.0)
No	2893 (96.9)	3863 (97.0)	6756(97.0)
High LDL-C			
Yes	68 (2.3)	76 (1.9)	144(2.1)
No	2918 (97.7)	3905 (98.1)	6823(97.9)
Low HDL-C			
Yes	1401 (46.9)	1518 (38.1)	2919(41.9)
No	1585 (53.1)	2463 (61.9)	4048(58.1)
Red meat intake			
> 100 g/day	1185 (39.7)	1115 (28.0)	2300(33.0)
≤ 100 g/day	1801 (60.3)	2866 (72.0)	4667(67.0)
Vegetables and fruits intake			
< 400 g/day	1515 (50.7)	1871 (47.0)	3386(48.6)
≥ 400 g/day	1471 (49.3)	2110 (53.0)	3581(51.4)
Fried food intake			
< 100 g/week	2366 (79.2)	3255 (81.8)	5621(80.7)
≥ 100 g/week	620 (20.8)	726 (18.2)	1346(19.3)
Sugar containing beverage intake			
< 300 ml/week	2395 (80.2)	3404 (85.5)	5799(83.2)
≥ 300 ml/week	591 (19.8)	577 (14.5)	1168(16.8)
Smoking			
Yes	2148 (71.9)	215 (5.4)	2363(33.9)
No	838 (28.1)	3766 (94.6)	4604(66.1)
Drinking			
Yes	1914 (64.1)	779 (19.6)	2693(38.6)
No	1072 (35.9)	3202 (80.4)	4274(61.4)
Sleep time, hours			
< 7	590 (19.8)	691 (17.4)	1281(18.4)
7 ~ 9	1887 (63.2)	2439 (61.3)	4326(62.1)
> 9	509 (17.0)	851 (21.4)	1360(19.5)
Sitting time, hours			
≤ 3	519 (17.4)	733 (18.4)	1252(18.0)
3.1 ~ 4	1201 (40.2)	1552 (39.0)	2753(39.5)
4.1 ~ 6	738 (24.7)	976 (24.5)	1714(24.6)
>6	528 (17.7)	720 (18.1)	1248(17.9)
Family history			
Yes	135 (4.5)	235 (5.9)	370(5.3)
No	2851 (95.5)	3746 (94.1)	6597(94.7)

### The incidence of DM according to different characteristics

Among the study subjects, there were 699 new diabetes patients (313 males and 386 females) newly diagnosed during the follow-up period, with a total follow-up of 40022.82 person-years (Table 3). The incidence density of diabetes was 17.5/1000 person-years. DM incidence density significantly differed according to age, marital status, region, weight status, central obesity status, hypertension status, dyslipidemia status, and family history of diabetes (all *P* < 0.05). There was no significant difference in the incidence density of diabetes according to sex, education level, urban or rural area, dietary intake, smoking status, drinking status, sleeping status or sitting status (all *P* > 0.05).

**Table 3:** The incidence of DM according to different characteristics

Characteristics	Number of DM	Follow-up person years	Incidence (/1000 person years)	*P*
All	699	40022.82	17.5	
Sex				0.28
Male	313	17091.75	18.3	
Female	386	22931.07	16.8	
Age, y				< 0.001
18 ~ 44	236	18049.02	13.1	
45 ~ 59	297	14907.27	19.9	
≥ 60	166	7066.53	23.5	
Marital status				< 0.001
Married	578	34180.55	16.9	
Unmarried	28	2277.51	12.3	
Divorce/Widow	93	3564.76	26.1	
Education level				0.147
≤Primary school	307	17295.81	17.7	
Secondary school	229	13707.02	16.7	
High school	120	5953.31	20.2	
≥College or above	43	3066.68	14	
Regions				0.01
East	142	6694.46	21.2	
Middle	346	19540.3	17.7	
West	211	13788.06	15.3	
Urban/rural				0.833
Urban	313	18060.98	17.3	
Rural	386	21961.84	17.6	
BMI				< 0.001
< 18.5	18	1608.36	11.2	
18.5 ~ 23.9	253	19483.35	13	
24 ~ 27.9	249	13181.9	18.9	
28.0~	179	5749.22	31.1	
Central obesity				< 0.001
Yes	429	30015.67	14.3	
No	270	10007.15	27	
Hypertension				< 0.001
No	327	24692.5	13.2	
Yes	372	15330.32	24.3	
Hypertriglyceridemia				< 0.001
Yes	141	4512.96	31.2	
No	558	35509.87	15.7	
Hypercholesterolemia				0.022
Yes	31	1182.25	26.2	
No	668	38840.57	17.2	
High LDL-C				0.017
Yes	23	804.1717194	28.6	
No	676	39218.65	17.2	
Low HDL-C				< 0.001
Yes	340	16656.64	20.4	
No	359	23366.18	15.4	
Red meat intake				0.054
> 100 g/day	208	13268.94	15.7	
≤ 100 g/day	491	26753.88	18.4	
Vegetables and fruits intake				0.412
< 400 g/day	350	19438.28	18	
≥ 400 g/day	349	20584.54	17	
Fried food intake				0.759
< 100 g/week	567	32296.12	17.6	
≥ 100 g/week	132	7726.7	17.1	
Sugar containing beverage intake				0.233
< 300 ml/week	593	33292.33	17.8	
≥ 300 ml/week	106	6730.49	15.7	
Smoking				0.795
Yes	234	13562.99	17.3	
No	465	26459.84	17.6	
Drinking				0.36
Yes	259	15483.42	16.7	
No	440	24539.41	17.9	
Sleep time, hours				0.266
< 7	142	7315.6	19.4	
7 ~ 9	432	24866.49	17.4	
> 9	125	7840.74	15.9	
Sitting time, hours				0.783
≤ 3	266	7198.59	37	
3.1 ~ 4	133	15825.49	8.4	
4.1 ~ 6	166	9866.18	16.8	
>6	136	7132.56	19.1	
Family history				< 0.001
No	643	37928.37	17	
Yes	56	2094.45	26.7	

### Risk factors for DM incidence based on Cox regression models

Cox regression model results for all the subjects, and for different gender groups were shown in Table 4. According to the Cox regression model results (Table 4), for all the populations, age 44 ~ 59 years (HR = 1.32, 95% CI: 1.10 ~ 1.59), age ≥ 60 years (HR = 1.48, 95% CI: 1.17 ~ 1.88), divorce/widowhood (HR = 1.45, 95% CI: 1.15 ~ 1.82), overweight but not central obesity (HR = 1.26, 95% CI: 1.03 ~ 1.54), overweight and central obesity (HR = 1.62, 95% CI: 1.34 ~ 1.97), hypertension (HR = 1.35, 95% CI: 1.15 ~ 1.59), hypertriglyceridemia (HR = 1.57, 95% CI: 1.28 ~ 1.91), high LDL-C (HR = 1.20, 95% CI: 1.02 ~ 1.40), and family history of DM (HR = 1.55, 95% CI: 1.17 ~ 2.06) were risk factors for diabetes.

For males, age ≥ 60 years (HR = 1.49, 95% CI: 1.07 ~ 2.08), divorce/widowhood (HR = 1.46, 95% CI: 1.01 ~ 2.10), overweight but not central obesity (HR = 1.47, 95% CI: 1.10 ~ 1.96), central obesity (HR = 3.34, 95% CI: 1.82 ~ 6.12), overweight and central obesity (HR = 1.76, 95% CI: 1.31 ~ 2.38), hypertension (HR = 1.36, 95% CI: 1.06 ~ 1.73), and sitting for > 6 h (HR = 1.51, 95% CI: 1.11 ~ 2.06) were risk factors. For females, age 45 ~ 59 years (HR = 1.44, 95% CI: 1.12 ~ 1.86), age ≥ 60 years (HR = 1.45, 95% CI: 1.03 ~ 2.02), divorce/widowhood (HR = 1.45, 95% CI: 1.07 ~ 1.96), overweight and central obesity (HR = 1.46, 95% CI: 1.14 ~ 1.87), hypertention (HR = 1.35, 95% CI: 1.07 ~ 1.69), hypertriglyceridemia (HR = 1.76, 95% CI: 1.34 ~ 2.31) and family history of DM (HR = 1.60, 95% CI: 1.11 ~ 2.30) were risk factors.

**Table 4:** The risk influencing factors of DM

Influencing factors	ALL		Male		Female	
	HR(95%CI)	*P*	HR(95%CI)	*P*	HR(95%CI)	*P*
Sex						
Male	1					
Female	0.82(0.66 ~ 1.01)	0.071	*—*		*—*	
Age, y						
18 ~ 44	1		1		1	
45 ~ 59	1.32(1.10 ~ 1.59)	0.003	1.19(0.91 ~ 1.57)	0.215	1.44(1.12 ~ 1.86)	0.006
≥ 60	1.48(1.17 ~ 1.88)	0.001	1.49(1.07 ~ 2.08)	0.022	1.45(1.03 ~ 2.02)	0.035
Marital status						
Married	1		1		1	
Unmarried	0.96(0.65 ~ 1.43)	0.847	0.78(0.47 ~ 1.31)	0.361	1.30(0.70 ~ 2.43)	0.424
Divorce/Widow	1.45(1.15 ~ 1.82)	0.002	1.46(1.01 ~ 2.10)	0.049	1.45(1.07 ~ 1.96)	0.019
Education level						
≤Primary school	1		1		1	
Secondary school	1.07(0.89 ~ 1.29)	0.474	0.96(0.72 ~ 1.28)	0.779	1.19(0.93 ~ 1.51)	0.174
High school	1.19(0.94 ~ 1.50)	0.149	1.22(0.88 ~ 1.69)	0.242	1.10(0.78 ~ 1.54)	0.583
≥College or above	0.90(0.63 ~ 1.28)	0.545	0.89(0.55 ~ 1.44)	0.636	0.85(0.49 ~ 1.47)	0.566
Regions						
East	1		1		1	
Middle	0.91(0.74 ~ 1.13)	0.398	0.86(0.62 ~ 1.19)	0.37	0.95(0.72 ~ 1.24)	0.701
West	0.83(0.66 ~ 1.05)	0.129	0.75(0.52 ~ 1.07)	0.119	0.88(0.64 ~ 1.19)	0.411
Urban/rural						
Urban	1		1		1	
Rural	1.05(0.89 ~ 1.25)	0.532	1.05(0.82 ~ 1.34)	0.722	1.08(0.86 ~ 1.35)	0.513
Overweight and obesity						
BMI < 24 and No central obesity	1		1		1	
BMI > 24 and No central obesity	1.26(1.03 ~ 1.54)	0.025	1.47(1.10 ~ 1.96)	0.01	1.08(0.82 ~ 1.42)	0.595
BMI < 24 and Central obesity	1.39(0.85 ~ 2.28)	0.203	3.34(1.82 ~ 6.12)	< 0.001	0.56(0.23 ~ 1.36)	0.205
BMI > 24 and Central obesity	1.62(1.34 ~ 1.97)	< 0.001	1.76(1.31 ~ 2.38)	< 0.001	1.46(1.14 ~ 1.87)	0.004
Hypertension						
No	1		1		1	
Yes	1.35(1.15 ~ 1.59)	0	1.36(1.06 ~ 1.73)	0.015	1.35(1.07 ~ 1.69)	0.011
Hypertriglyceridemia						
No	1		1		1	
Yes	1.57(1.28 ~ 1.91)	< 0.001	1.30(0.97 ~ 1.75)	0.081	1.76(1.34 ~ 2.31)	< 0.001
Hypercholesterolemia						
No	1		1		1	
Yes	0.89(0.55 ~ 1.44)	0.615	0.54(0.24 ~ 1.25)	0.16	1.19(0.66 ~ 2.15)	0.613
High LDL-C						
No	1		1		1	
Yes	1.20(1.02 ~ 1.40)	0.03	1.25(0.98 ~ 1.60)	0.075	1.14(0.92 ~ 1.40)	0.261
Low HDL-C						
No	1		1		1	
Yes	1.40(0.80 ~ 2.42)	0.241	2.18(0.98 ~ 4.86)	0.061	1.03(0.48 ~ 2.23)	0.917
Red meat intake						
≤ 100 g/day	1		1		1	
> 100 g/day	1.00(0.84 ~ 1.18)	0.974	1.06(0.83 ~ 1.34)	0.636	0.95(0.74 ~ 1.22)	0.692
Smoking						
No	1		1		1	
Yes	0.89(0.71 ~ 1.10)	0.274	0.90(0.70 ~ 1.16)	0.418	0.85(0.53 ~ 1.36)	0.498
Drinking						
No	1		1		1	
Yes	0.91(0.76 ~ 1.09)	0.315	0.97(0.76 ~ 1.23)	0.807	0.85(0.64 ~ 1.12)	0.264
Sleeping time, hours						
< 7	1		1		1	
7 ~ 9	1.02(0.84 ~ 1.24)	0.846	1.31(0.96 ~ 1.78)	0.095	0.85(0.66 ~ 1.10)	0.226
> 9	0.97(0.76 ~ 1.25)	0.819	1.36(0.92 ~ 2.00)	0.128	0.76(0.55 ~ 1.06)	0.111
Sitting time, hours						
≤ 3	1		1		1	
3.1 ~ 4	0.93(0.75 ~ 1.15)	0.501	0.92(0.66 ~ 1.27)	0.601	0.92(0.70 ~ 1.21)	0.575
4.1 ~ 6	0.96(0.79 ~ 1.17)	0.714	1.00(0.74 ~ 1.35)	0.989	0.95(0.73 ~ 1.23)	0.679
>6	1.11(0.89 ~ 1.38)	0.372	1.51(1.11 ~ 2.06)	0.01	0.80(0.58 ~ 1.10)	0.183
Family history						
No	1		1		1	
Yes	1.55(1.17 ~ 2.06)	0.002	1.46(0.93 ~ 2.27)	0.103	1.60(1.11 ~ 2.30)	0.012

Note: The models included sex, age, marital status, education level, region, urban and rural area, overweight and obesity status, hypertension status, dyslipidemia status, current smoking status, current drinking status, sleep duration, sitting time, excessive red meat intake, and family history of diabetes

### Sensitivity analysis

Sensitivity analysis results (Table 5) showed that BMI > 24 kg/m^2^ and no central obesity were no longer significant among the total participants. In the male participants, the Divorce/Widow variable was no longer significant, and hypertriglyceridemia, high LDL-C, low HDL-C, and family history became significant. The sensitivity results showed that the associations between the influencing factors and DM did not change substantially and validated the associations in the preceding text.

**Table 5:** Sensitivity analysis for DM and influencing factors

Influencing factors	ALL		Male		Female	
	HR(95%CI)	*P*	HR(95%CI)	*P*	HR(95%CI)	*P*
**Sex**						
Male						
Female	0.77(0.61 ~ 0.96)	0.022	*—*		*—*	
Age, y						
18 ~ 44	1		1		1	
45 ~ 59	1.35(1.11 ~ 1.64)	0.003	1.22(0.92 ~ 1.63)	0.172	1.43(1.10 ~ 1.87)	0.008
≥ 60	1.56(1.22 ~ 2.00)	0	1.61(1.14 ~ 2.27)	0.007	1.47(1.03 ~ 2.08)	0.032
Marital status						
Married	1		1		1	
Unmarried	0.97(0.64 ~ 1.46)	0.868	0.86(0.51 ~ 1.43)	0.557	1.28(0.66 ~ 2.46)	0.463
Divorce/Widow	1.46(1.15 ~ 1.86)	0.002	1.37(0.94 ~ 2.00)	0.104	1.45(1.06 ~ 1.98)	0.02
Education level						
≤Primary school	1		1		1	
Secondary school	1.07(0.88 ~ 1.30)	0.489	1.00(0.75 ~ 1.34)	0.99	1.20(0.93 ~ 1.54)	0.159
High school	1.18(0.93 ~ 1.51)	0.173	1.16(0.83 ~ 1.63)	0.391	1.15(0.81 ~ 1.64)	0.422
≥College or above	0.81(0.55 ~ 1.19)	0.283	0.88(0.53 ~ 1.45)	0.62	0.80(0.45 ~ 1.42)	0.448
Regions						
East	1		1		1	
Middle	0.91(0.73 ~ 1.14)	0.422	0.89(0.63 ~ 1.26)	0.523	0.89(0.67 ~ 1.18)	0.42
West	0.87(0.68 ~ 1.12)	0.279	0.80(0.54 ~ 1.17)	0.247	0.87(0.63 ~ 1.20)	0.397
Urban/rural						
Urban	1		1		1	
Rural	1.03(0.86 ~ 1.23)	0.739	1.11(0.86 ~ 1.44)	0.408	1.01(0.80 ~ 1.28)	0.929
Overweight and obesity						
BMI < 24 and No central obesity	1		1		1	
BMI > 24 and No central obesity	1.23(1.00 ~ 1.51)	0.056	1.40(1.04 ~ 1.89)	0.026	1.06(0.79 ~ 1.40)	0.707
BMI < 24 and Central obesity	1.05(0.60 ~ 1.85)	0.867	2.54(1.28 ~ 5.02)	0.007	0.44(0.16 ~ 1.21)	0.113
BMI > 24 and Central obesity	1.55(1.27 ~ 1.90)	< 0.001	1.60(1.18 ~ 2.18)	0.003	1.42(1.09 ~ 1.84)	0.009
Hypertension						
No	1		1		1	
Yes	1.32(1.11 ~ 1.57)	0.002	1.38(1.07 ~ 1.77)	0.013	1.36(1.08 ~ 1.72)	0.009
Hypertriglyceridemia						
No	1		1		1	
Yes	1.55(1.26 ~ 1.91)	< 0.001	1.38(1.02 ~ 1.86)	0.039	1.70(1.28 ~ 2.26)	0
Hypercholesterolemia						
No	1		1		1	
Yes	1.00(0.61 ~ 1.65)	0.991	0.56(0.24 ~ 1.31)	0.18	1.33(0.73 ~ 2.42)	0.345
High LDL-C						
No	1		1		1	
Yes	1.17(0.99 ~ 1.39)	0.059	1.29(1.00 ~ 1.66)	0.049	1.11(0.89 ~ 1.38)	0.364
Low HDL-C						
No	1		1		1	
Yes	1.40(0.79 ~ 2.48)	0.245	2.35(1.04 ~ 5.30)	0.04	1.07(0.50 ~ 2.32)	0.858
Red meat intake						
≤ 100 g/day	1		1		1	
> 100 g/day	1.18(0.94 ~ 1.49)	0.659	1.05(0.82 ~ 1.35)	0.691	0.99(0.77 ~ 1.28)	0.94
Smoking						
No	1		1		1	
Yes	0.86(0.69 ~ 1.08)	0.197	0.88(0.68 ~ 1.13)	0.318	0.84(0.51 ~ 1.39)	0.503
Drinking						
No	1		1		1	
Yes	0.90(0.75 ~ 1.09)	0.288	1.00(0.78 ~ 1.29)	0.991	0.86(0.64 ~ 1.14)	0.292
Sleeping time, hours						
< 7	1		1		1	
7 ~ 9	1.10(0.90 ~ 1.36)	0.358	1.32(0.96 ~ 1.81)	0.091	0.89(0.68 ~ 1.16)	0.372
> 9	0.98(0.75 ~ 1.28)	0.906	1.35(0.90 ~ 2.03)	0.144	0.76(0.54 ~ 1.07)	0.118
Sitting time, hours						
≤ 3	1		1		1	
3.1 ~ 4	0.97(0.78 ~ 1.21)	0.766	0.92(0.66 ~ 1.30)	0.645	0.98(0.74 ~ 1.29)	0.862
4.1 ~ 6	0.98(0.80 ~ 1.21)	0.87	0.99(0.72 ~ 1.36)	0.962	0.95(0.73 ~ 1.25)	0.722
>6	1.18(0.94 ~ 1.49)	0.154	1.63(1.19 ~ 2.24)	0.003	0.86(0.62 ~ 1.19)	0.364
Family history						
No	1		1		1	
Yes	1.67(1.25 ~ 2.24)	0.001	1.58(1.01 ~ 2.48)	0.046	1.71(1.18 ~ 2.49)	0.005

## Discussion

China has the largest DM population in the world, and the burden continues to increase, resulting in not only great health damage, life losses but also heavy economic burdens. According to the estimated results of the IDF on the incidence rate and incidence trend of diabetes in various countries (regions), the number of diabetes patients in China reached 116.4 million in 2019, ranking first in the world, and this trend is predicted to continue until 2045 [2]. The estimated number of deaths from DM in China in 2020 was 253,968 persons, and the estimated years of life lost (YLL) was 4,929,541.12 × 100,000 person-years [22]. China has the largest health expenditure on diabetes other than that of United States of America(USA), reaching $165.3 billion in 2021 [2]. Wide attention has been given to DM in China, and many studies have been conducted in China to explore the prevalence and influencing factors of DM [4, 5, 23]. However, due to the fact that the prevalence of DM is affected by both the risk of developing DM and the survival of those with DM, the incidence of DM is a better metric for understanding trends in the population risk of DM [24]. Exploring the incidence of DM and its influencing factors could provide scientific data and scientific support for the formulation of relevant policies for timely mastering of the incidence data and influencing factors of DM in China. This study took advantage of the national representative surveillance database and designed a follow-up study. Based on the follow-up results, the incidence of DM and related influencing factors were analyzed, which could provide important information on DM incidence in China and help to identify key influencing factors.

The results of this study showed that the incidence density of DM in China is 17.5/1000 person-years. According to a systematic review, the incidence of diagnosed DM is stabilizing or declining in many high-income countries [25], while in China, the estimated DM incidence was high and increased from 2013 to 2018 [5]. More work should be done on DM surveillance to grasp the trend of diabetes development and changes in China.

The results of this study showed that age 45 ~ 59 years (HR = 1.32, 95% CI: 1.10 ~ 1.59) and age ≥ 60 years (HR = 1.48, 95% CI: 1.17 ~ 1.88) were risk factors for DM, which was consistent with the findings of other studies in China [6, 8]. Aging increases the risk of metabolic syndrome and chronic diseases, including type 2 DM [14]. Divorce/widowhood (HR = 1.45, 95% CI: 1.15–1.82) was found to be a risk factor for DM in this study. Marital relationships could be causally linked to type 2 DM risk and worsening glycemic regulation through stress buffering mechanisms, social regulation processes, and socioeconomic pathways [26–29]. A family history of diabetes (HR = 1.55, 95% CI: 1.17 ~ 2.06) was a risk factor for DM, which was consistent with the findings of large studies in China [8]. Family history reflects both genetic and environmental factors [14].

Obesity has long been recognized as an important risk factor for DM [30, 31]. In this study, BMI > 24.0 kg/m^2^ (for non-central obesity: HR = 1.26, 95% CI: 1.03 ~ 1.54; for central obesity: HR = 1.62, 95% CI: 1.34 ~ 1.97) was a risk factor for DM. This finding was consistent with the results of large-scale studies in China [8], and a nationally representative survey of chronic diseases and their risk factors among Chinese residents [5]. Both overweight and central obesity increased the risk of DM in males (HR = 1.47–3.34, *P* < 0.001), and the population with the highest risk was the male population with only central obesity (HR = 3.34, 95% CI: 1.82 ~ 6.12). For females, both overweight and central obesity increased the risk of DM (HR = 1.46, 95% CI: 1.14–1.87), which indicated that for the female population, both overweight and central obesity should be considered when determining the risk of diabetes. Hypertension (HR = 1.35, 95% CI: 1.15 ~ 1.59) and dyslipidemia were also found to be risk factors for diabetes, which was consistent with the results of large national studies [8] and other studies [32, 33]. Hypertriglyceridemia and high LDL-C were found to increase DM risk in this study. As to life styles, for males, sitting for > 6 h was a risk factor, which was consistent with the findings of other studies [34]. With rapid economic growth and associated industrialization, urbanization, and lifestyle changes (increased consumption of high-calorie, high-fat, high-sugar, and high-sodium diets and decreased physical activity), overweight and obesity, hypertension, hyperlipidemia, and hyperglycemia have reached high epidemic proportions in the Chinese population, and the prevention and control of DM in China cannot be separated from the control of these related risk factors [6].

The results of this study indicated that keeping a healthy diet, taking adequate physical exercises, and maintaining a healthy weight should be further implemented in China to prevent the DM. Though previous studies and evidences have advocated these control measures related to the risking factors of DM, the effect is still not ideal and the incidence of DM in China is still rising, which indicated that the preventive and control measures for DM have not been effectively implemented. In addition, due to the vast territory and the imbalance in economic and medical development of China, the allocation of prevention and control resources is uneven, and the service capacity at the grassroots level needs to be improved [35]. Nevertheless, China has proposed the national action plan of “Healthy China 2030” [36], which aims to effectively control the incidence and mortality rates of chronic diseases through policy support, technological means, and social participation. The prevention and control of DM has been prioritised as one of the 15 special campaigns (and one of the four chronic diseases) to achieve a healthy China by 2030 [36]. And, the multi-level and multi-departmental collaborative system for chronic disease prevention and control has been established and is continuously evolving, which would provide solid resource support for the prevention and control of DM in different regions of China.

There were several limitations of this study. There was some loss to follow-up populations, and there were significant differences between the missing and follow-up populations in this study, which may have biased the results. Due to the many factors affecting DM, the baseline survey did not collect all exposure information such as detailed dietary habits, socioeconomic (e.g., income, employment, social relationships) and psychological factors (such as hormone level, anxiety and depression), and environmental factors (e.g., air pollution, endocrine disrupting chemicals) [6, 37, 38], which may have led to residual confounding. In future studies, we will take into account a broader range of factors and promote interdisciplinary integration to interpret disparities of DM incidences. In addition, during the follow-up period, some influencing factors such as the marital status and lifestyles might have changed comparing with the baseline status, and the changed influencing factors may affect the onset of DM, which may also have biased the results.

## Conclusion

Among the 7048 people studied, 699 had diabetes (313 males and 386 females), with a total follow-up of 40022.82 person-years. The incidence density of diabetes was 17.5/1000 person-years. The Cox regression models showed that age > 44 years, divorce/widowhood, overweight status, central obesity status, hypertension status, dyslipidemia status, and diabetes family history were risk factors for diabetes in the Chinese population. Comprehensive preventive measures centered on a healthy diet, adequate physical exercise, and maintaining a healthy weight should be further promoted to prevent diabetes in China.

## Data Availability

No datasets were generated or analysed during the current study.

## Abbreviations

BMI: Body Mass Index
BRICS: Brazil, Russia, India, China, and South Africa
CCDRFS: China Chronic Disease and Risk Factors Surveillance
CIs: confidence intervals
DBP: Diastolic Blood Pressure
DM: Diabetes mellitus
FPG: Fasting blood glucose
HDL-C: High-density lipoprotein cholesterol
HRs: Hazard ratios
IDF: International Diabetes Federation
LDL-C: Low-density lipoprotein cholesterol
NBS: National Bureau of Statistics
SBP: Systolic Blood Pressure
TC: Total Cholesterol
T2DM: Type 2 diabetes mellitus
TG: Triglyceride
USA: United States of America
WHO: World Health Organization
YLL: Years of life lost
